# Enhancing Image–Text Retrieval via Region–Grid Interaction and Semantic Calibration

**DOI:** 10.3390/s26144483

**Published:** 2026-07-15

**Authors:** Can Lu, Muye Feng

**Affiliations:** School of Mechanical and Power Engineering, Nanjing Tech University, Nanjing 211816, China; lc123@njtech.edu.cn

**Keywords:** image–text retrieval, region–grid fusion, semantic-guided interaction, adaptive feature fusion

## Abstract

Image–Text retrieval requires accurate semantic alignment between visual content and natural language descriptions. Most existing methods primarily rely on region features, which are typically object-centric and may overlook important contextual information. In contrast, grid features provide denser spatial coverage and richer local details, but often lack explicit semantic structure. To better exploit their complementarity, we propose a novel Region–Grid Interaction and Calibration Network (RGICN) for Image–Text retrieval. Specifically, we first design a Global-Guided Feature Interaction Module to promote information exchange between region and grid features under the guidance of global visual semantics, allowing object-level semantics and contextual cues to complement each other. We then introduce a Text-Guided Feature Calibration Module, which leverages auxiliary image descriptions to calibrate visual features by suppressing redundant and text-irrelevant content. Finally, an Adaptive Gating Fusion Module is developed to dynamically integrate multiple visual representations according to the input image, yielding a more comprehensive and discriminative visual embedding. Extensive experiments on the benchmark datasets MS-COCO and Flickr30K demonstrate the effectiveness of RGICN and its competitive performance against recent state-of-the-art methods.

## 1. Introduction

**Figure 1:**
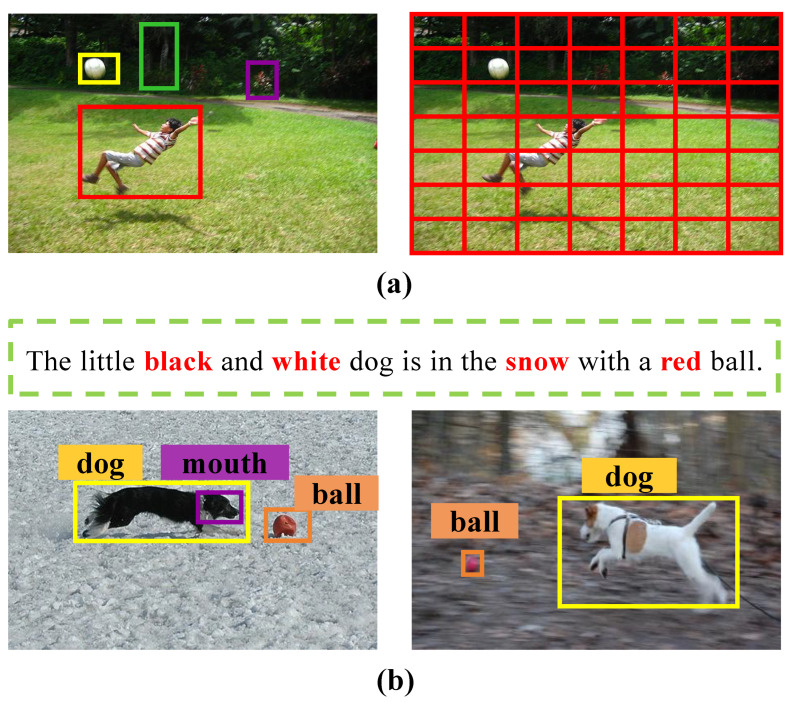
(**a**) Comparison between region features (**left**) and grid features (**right**). Region features focus on salient objects and semantic concepts, whereas grid features preserve dense visual information over the whole image. (**b**) Due to their inherent limitations, region features may fail to capture fine-grained object details and global contextual cues, making it challenging for the model to discriminate between visually similar images and thus causing retrieval errors. From top to bottom, we show the query sentence, the ground-truth image (**left**), and the incorrectly retrieved image (**right**).

Image–Text retrieval aims to retrieve the most semantically relevant images for a given textual query, and vice versa. As a representative task in cross-modal learning, it plays an important role in numerous real-world applications, such as image captioning, person re-identification, and cross-modal retrieval. Despite the remarkable progress achieved in recent years, Image–Text retrieval remains highly challenging due to the inherent heterogeneous gap between visual and textual modalities. In recent years, Image–Text retrieval [1,2,3,4,5] has attracted substantial research attention, with modality interaction modeling and similarity estimation being the primary focus of existing studies. For example, SCAN [5] adopts a stacked cross-attention network to capture fine-grained interactions between image regions and words, while GRAN [6] models intra-modal local relationships and local-global interactions via global and relation-aware attention modules. ESL [7] further proposes an enhanced semantic similarity learning framework to learn multi-dimensional alignments between visual and textual features. Although these methods have achieved promising results, most of them still rely primarily on region features [8] to represent images. However, region features extracted by object detectors [9] are inherently object-centric and may fail to provide complete coverage of the entire image, leading to the omission of small objects and the loss of global contextual information, as illustrated in Figure 1.

To overcome this limitation, recent studies [10,11,12,13,14] have explored the use of grid features extracted from CNNs or Vision Transformers [15,16]. Compared with region features, grid features provide denser spatial coverage over the whole image and are therefore able to preserve richer contextual cues and finer local details. Nevertheless, unlike semantically complete object regions, grid features are usually defined on uniform spatial partitions and may not align well with meaningful semantic entities [10]. As a result, although grid features are informative in terms of spatial detail and contextual completeness, they often suffer from weak semantic coherence and may introduce ambiguity into visual representation learning. From this perspective, region and grid features exhibit highly complementary properties: the former emphasizes object-level semantics, while the latter captures holistic scene context and fine-grained details. Therefore, effectively integrating these two types of visual features is expected to yield more powerful and informative image representations for cross-modal matching.

**Figure 2:**
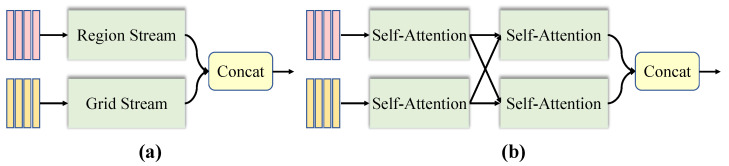
Illustration of common fusion strategies for region and grid features: (**a**) a dual-stream strategy that processes region and grid features independently and concatenates their outputs; and (**b**) a cross-stream interaction strategy that enables information exchange between region and grid features before concatenation.

To this end, some existing methods [17] adopt a dual-stream architecture to process region features and grid features in parallel, and then simply concatenate the outputs of the two branches to form the final visual representation, as illustrated in Figure 2a. Although such a design allows the model to exploit both types of features, it treats them largely as independent visual sources and does not explicitly model their semantic interactions. As a result, the complementary information between object-centric regions and spatially dense grids cannot be fully exploited. To address this issue, other methods [18,19] attempt to enhance one type of feature by directly aggregating information from the other through a cross-attention mechanism. Specifically, region features can be refined by attending to all grid features, or conversely, grid features can be updated by incorporating information from all region features, as shown in Figure 2b. While these strategies introduce cross-feature interaction, directly aggregating information from all features may also inject redundant, noisy, or less relevant content into the learned representations. This issue is particularly severe in Image–Text retrieval, where only part of the visual content is truly relevant to the accompanying text, while the rest may correspond to background context or unrelated objects.

Therefore, an important challenge is to effectively exploit the complementarity between region and grid features while reducing the interference caused by redundant or text-irrelevant visual information. To this end, we propose a Region–Grid Interaction and Calibration Network (RGICN) for Image–Text retrieval. Specifically, RGICN first employs a Global-Guided Feature Interaction Module to enable bidirectional interaction between region-level and grid-level features under the guidance of global visual semantics, so that object-level semantics and contextual cues can be mutually enhanced. To further improve the semantic quality of visual representations, we introduce a Text-Guided Feature Calibration Module, which uses auxiliary image descriptions generated by a large vision-language model (e.g., LLaVA) as semantic priors to calibrate visual features by suppressing redundant and text-irrelevant content. Since the relative importance of region and grid representations varies across images, an Adaptive Gating Fusion Module is further designed to dynamically balance their contributions and generate the final visual embedding. In this way, RGICN integrates region–grid interaction, Text-Guided semantic calibration, and instance-adaptive fusion into a unified framework, producing more comprehensive and discriminative visual representations for Image–Text retrieval. In summary, the main contributions of this paper are as follows:

- We propose a Region–Grid Interaction and Calibration Network for Image–Text retrieval, which exploits the complementary properties of region and grid features to learn more informative visual representations for cross-modal matching.
- To effectively integrate region and grid features, we design a unified interaction and calibration framework, including a global-guided feature interaction mechanism for bidirectional information exchange and a Text-Guided calibration and adaptive fusion strategy for enhancing feature complementarity and suppressing redundant or text-irrelevant information.
- Extensive experiments on MS-COCO and Flickr30K demonstrate the effectiveness of the proposed method and show that it achieves superior performance compared with recent state-of-the-art approaches.

## 2. Related Work

### 2.1. Image–Text Retrieval

With the rapid development of deep learning techniques, Image–Text retrieval has achieved remarkable progress. The core challenge of this task lies in bridging the heterogeneous gap between image and text modalities, so as to ensure semantic consistency between Image–Text pairs. According to the alignment strategy, existing Image–Text retrieval methods [1,2,10,17,20,21,22,23,24] can be roughly divided into two categories: one-to-one global alignment and many-to-many local alignment.

Global alignment methods mainly focus on mapping the entire image and sentence into a shared embedding space, where their similarity can be directly computed based on the cosine distance between the global embeddings. Considerable progress has been made in this line of research, ranging from the design of novel loss functions, to the development of specialized feature extraction architectures for different models, and to the learning of better pooling strategies. For example, VSE++ [3] introduced a triplet loss with hard negative mining to pull matched Image–Text pairs closer while pushing mismatched pairs farther apart, which has been widely adopted by subsequent studies. GPO [25] proposed a generalized pooling operator that can adaptively learn the optimal feature aggregation strategy for different data. USER [26] carefully designed two simple yet effective semantic enhancement modules based on global representations, and further introduced a momentum contrastive learning paradigm, which effectively enlarges the scale of negative samples during training. SSCT [27] designed a similarity-shuffled criss-cross Transformer to highlight salient features and relevant data, which helps generate sparse similarity maps, and further proposed a novel angle loss to enlarge the distance between positive and negative samples.

Local alignment methods aim to explore the complex interactions between image regions and textual words, and then aggregate these local matches into a global relevance score. Different from global alignment methods, these approaches explicitly model fine-grained semantic correspondences between local features, thereby achieving more accurate retrieval performance. As a pioneering work, SCAN [5] employed a stacked cross-attention network to comprehensively discover fine-grained alignments between image regions and textual words. Building upon this framework, a series of methods [1,20,28,29,30] have been proposed. For instance, NAAF [29] leveraged both matched and mismatched local alignments to infer Image–Text similarity. CHAN [28] accurately discovered shared semantics between images and text by mining the most relevant region-word pairs and removing redundant or irrelevant alignments based on a hard assignment encoding mechanism. Hire [31] models both intra-modal and inter-modal semantic relationships among image regions and textual words through implicit and explicit relation modeling. CMCL [32] used semantic prototypes as a bridge and employed a semantic decoder to attend to semantically consistent content across different modalities, thereby naturally achieving cross-modal semantic alignment. Inspired by human cognitive mechanisms, CMSF [33] enriched cross-modal interactions through a spike-level fusion mechanism and combined it with a dual-stream architecture to achieve efficient and effective Image–Text retrieval.

Although existing methods have achieved significant progress, they usually rely on only a single type of visual feature to represent an image, making it difficult to comprehensively capture the rich information contained in visual content. To address this issue, we propose to represent images using both region features and grid features, and to obtain a more comprehensive and profound understanding of image content through deep fusion between them. In this way, the feature representation capability of the model can be further enhanced, leading to improved performance on Image–Text retrieval.

### 2.2. Visual Representations

Learning comprehensive and discriminative visual representations is crucial for Image–Text retrieval. With the rapid development of computer vision, the visual representations used in this task have evolved through three major stages.

In the first stage, CNN-based models, such as VGG and ResNet, were widely adopted to extract global image features. Although these holistic representations are compact and efficient, their fixed-length nature inevitably results in information loss and limited semantic granularity [34]. In the second stage, with the emergence of R-CNN-based object detectors and advances in related vision-language tasks, visual representations gradually shifted from CNN-derived grid features to detector-extracted region features. Compared with grid features, region features provide object-level semantics that are critical for understanding complex visual scenes, and thus became the dominant choice for Image–Text retrieval. However, region features also suffer from inherent limitations, including insufficient contextual information and the lack of fine-grained visual details. To overcome these issues, recent methods have revisited grid features extracted by Vision Transformers, marking the transition to the third stage. Following this trend, a number of studies have explored transformer-based grid features for Image–Text retrieval. For example, LAPS [10] explicitly identifies redundant visual patches with language supervision and rectifies their semantic and spatial information to facilitate more consistent fine-grained alignment. GRM [35] proposes a significance- and granularity-aware adaptive approach to model intra-modal biases, suppress redundant information, and improve cross-modal alignment generalization. PICO [14] reduces the weights of feature dimensions dominated by style information during embedding interaction, thereby mitigating information bias and preventing feature loss. CDDS [12] proposes a constrained decoupling and distribution sampling method to disentangle semantic and modality-specific components for cross-modal alignment. Overall, the continuous advancements in multimodal algorithms alongside the integration of multi-level feature representations [36,37,38,39] provide profound inspiration and valuable reference for addressing the complexities of this task. Furthermore, beyond these classical deep learning architectures, emerging research has recently begun to explore quantum computing [40,41] as a groundbreaking paradigm for visual representation. By leveraging quantum mechanical properties such as superposition and entanglement, quantum-enhanced vision models exhibit the theoretical potential to capture highly complex spatial correlations and provide exponentially larger state spaces for feature encoding.

In this paper, we investigate how to integrate region and grid features more effectively. By jointly modeling cross-feature interaction, semantic calibration, and instance-adaptive fusion, our method learns more comprehensive and discriminative visual representations, leading to significant improvements in Image–Text retrieval performance.

## 3. Methods

**Figure 3:**
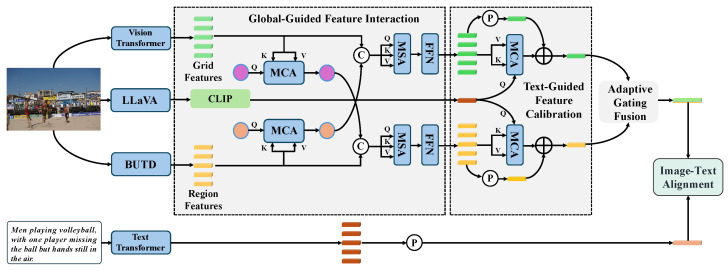
Illustration of our proposed framework. For an input image, we first extract region features and grid features using the BUTD model and a Vision Transformer, respectively. Then, the proposed Global-Guided Feature Interaction Module is employed to enhance the complementarity between the two types of features, while the Text-Guided Feature Calibration Module is used to remove redundant visual information. Finally, an Adaptive Gating Fusion Module is introduced to obtain a comprehensive and discriminative global visual representation. For an input text, we use a text transformer to extract word representations, followed by a pooling operation to generate the global textual representation. We adopt a hinge-based triplet ranking loss to train the entire model for Image–Text alignment.

In this section, we provide a detailed introduction to the proposed feature fusion network, as illustrated in Figure 3. We begin by extracting and encoding the initial textual and visual features, including both region-level and grid-level representations. We then present the Global-Guided Feature Interaction Module, the Text-Guided Feature Calibration Module, and the Adaptive Gating Fusion Module. These modules are designed to model the interactions between region and grid features, suppress redundant and text-irrelevant visual information, and adaptively balance complementary visual cues, respectively. Through these designs, the model is able to learn more comprehensive and discriminative visual features. Finally, we introduce the objective function used for model training and optimization.

### 3.1. Feature Extraction

#### 3.1.1. Region Feature Extraction

We employ the bottom-up top-down attention (BUTD) model [8] to extract region features. Specifically, a pretrained Faster R-CNN detector [9] is first used to generate $N_{r}$ region proposals for each image. Then, a ResNet-101 network [42] is applied to extract a feature vector for each region, denoted as $r_{i}\in R^{d_{r}}$, where $d_{r}$ is the feature dimension. To project the region features into a shared *d*-dimensional embedding space, we further apply a fully connected layer followed by a two-layer multi-layer perceptron (MLP) with a residual connection:

(1) $$v_{i}=\mathrm{MLP}\left(r_{i}\right)+f_{i},\;f_{i}=W^{r}r_{i}+b^{r},$$

where $W^{r}\in R^{d\times d_{r}}$ and $b^{r}\in R^{1\times d}$ denote the weight matrix and bias vector of the fully connected layer, respectively. The resulting region feature set is denoted as $R=\{v_{1},v_{2},\ldots ,v_{N_{r}}|v_{i}\in R^{d}\}$.

#### 3.1.2. Grid Feature Extraction

We employ a Vision Transformer, e.g., ViT [15] or Swin Transformer [16], to extract grid features. Specifically, the input image is first divided into $N_{g}$ non-overlapping patches. These patches are then flattened and linearly projected into a sequence of patch embeddings, which are subsequently fed into a multi-layer transformer encoder to capture dependencies among different image patches. In this way, a feature vector is obtained for each patch, denoted as $g_{i}\in R^{d_{g}}$, where $d_{g}$ is the feature dimension. Each grid feature $g_{i}$ is then projected into the same *d*-dimensional space through a fully connected layer:

(2) $$u_{i}=W^{g}g_{i}+b^{g},$$

where $W^{g}\in R^{d\times d_{g}}$ and $b^{g}\in R^{1\times d}$ denote the weight matrix and bias vector, respectively. The resulting grid feature set is denoted as $G=\{u_{1},u_{2},\ldots ,u_{N_{g}}|u_{i}\in R^{d}\}$.

#### 3.1.3. Textual Feature Extraction

We adopt a sequence transformer, i.e., BERT [43], as the textual backbone to extract token-level textual features. Specifically, the input sentence is first tokenized into $N_{t}$ subword tokens, which are then fed into a multi-layer transformer encoder to obtain contextualized textual representations. Accordingly, a feature vector is extracted for each token, denoted as $t_{i}\in R^{d_{t}}$, where $d_{t}$ is the feature dimension. Each textual feature $t_{i}$ is further projected into the shared *d*-dimensional space through a fully connected layer:

(3) $$h_{i}=W^{t}t_{i}+b^{t},$$

where $W^{t}\in R^{d\times d_{t}}$ and $b^{t}\in R^{1\times d}$ denote the weight matrix and bias vector, respectively. The resulting textual feature set is denoted as $H=\{h_{1},h_{2},\ldots ,h_{N_{t}}|h_{i}\in R^{d}\}$. Finally, a pooling operation is applied to the token-level textual features to obtain the global textual representation $t\in R^{d}$.

### 3.2. Global-Guided Feature Interaction Module

Region features and grid features are inherently complementary. Specifically, region features mainly provide object-centric information, while grid features capture richer contextual semantics. Integrating these two types of visual features can therefore yield a more comprehensive image representation. However, simple feature fusion is insufficient to fully exploit their complementary strengths, as it lacks explicit interaction between heterogeneous features. To address this issue, we propose a Global-Guided Feature Interaction Module (GFIM), which introduces global tokens to facilitate message passing across different feature types, thereby enabling deeper interaction and integration between region and grid features.

In implementation, the proposed GFIM is composed of *N* stacked identical blocks. In the (l+1)-th block, given the input region features $V_{R}^{l}$ and grid features $V_{G}^{l}$, we first introduce two learnable global tokens, denoted as $c_{r}^{l}$ and $c_{g}^{l}$, respectively. Subsequently, to capture global contextual information within each visual feature stream, these two global tokens are fed into a multi-head cross-attention module (MCA) as queries, while the corresponding feature sets are used as keys and values. Through this operation, each token selectively aggregates informative global cues from its associated feature stream, thereby enhancing the global summarization ability of region and grid representations. The process can be formulated as follows:

(4) $${c^{\prime }}_{r}^{l+1}=\mathrm{MCA}\left(c_{r}^{l},V_{R}^{l},V_{R}^{l}\right),$$

(5) $${c^{\prime }}_{g}^{l+1}=\mathrm{MCA}\left(c_{g}^{l},V_{G}^{l},V_{G}^{l}\right),$$

where MCA(·) denotes the multi-head cross-attention operation. Following the standard multi-head attention formulation, its computation can be expressed as follows:

(6) $$\mathrm{MCA}\left(Q,K,V\right)=\mathrm{Concat}\left({\mathrm{head}}_{1},\ldots ,{\mathrm{head}}_{H}\right)W^{O},$$

(7) $${\mathrm{head}}_{i}=\mathrm{Attention}\left(QW_{i}^{Q},KW_{i}^{K},VW_{i}^{V}\right),$$

(8) $$\mathrm{Attention}\left(Q,K,V\right)=\mathrm{Softmax}\left(\frac{QK^{⊤}}{\sqrt{d}}\right)V,$$

where Q, K, and V denote the query, key, and value matrices, respectively. *H* denotes the number of attention heads, and ${\mathrm{head}}_{i}$ represents the output of the *i*-th attention head. $W_{i}^{Q}$, $W_{i}^{K}$, and $W_{i}^{V}$ are the learnable projection matrices for the query, key, and value in the *i*-th head, respectively, while $W^{O}$ is the output projection matrix. *d* denotes the feature dimension of each attention head, and Softmax(·) is used to normalize the attention scores.

Afterward, the two globally enhanced tokens are exchanged and concatenated with the opposite feature streams. Specifically, the global token refined from the region-level feature stream is appended to the grid-level features, while the global token refined from the grid-level feature stream is appended to the region-level features. The resulting feature sequences are then fed into two separate multi-head self-attention (MSA) modules. On the one hand, the MSA modules capture intra-feature dependencies within each feature stream. On the other hand, by virtue of the exchanged global tokens, they also enable implicit cross-feature information propagation, thereby further aligning and integrating the region and grid representations. This process is formulated as follows:

(9) $${V^{\prime }}_{R}^{l+1}=\mathrm{MSA}\left(V_{R}^{l},\left[V_{R}^{l},{c^{\prime }}_{g}^{l+1}\right],\left[V_{R}^{l},{c^{\prime }}_{g}^{l+1}\right]\right),$$

(10) $${V^{\prime }}_{G}^{l+1}=\mathrm{MSA}\left(V_{G}^{l},\left[V_{G}^{l},{c^{\prime }}_{r}^{l+1}\right],\left[V_{G}^{l},{c^{\prime }}_{r}^{l+1}\right]\right),$$

where MSA(·) follows the same computation as MCA(·) under the standard multi-head attention formulation, but differs in its input construction: the query is taken from the current feature stream, while the key and value are formed by concatenating that stream with the exchanged global token.

Finally, a feed-forward network (FFN) is applied to further transform and refine the output representations. Similar to the attention sublayers, the FFN is followed by a residual connection and a layer normalization operation, which help preserve the original information, stabilize the training process, and facilitate the optimization of the overall architecture. The corresponding operations are defined as follows:

(11) $$V_{R}^{l+1}=\mathrm{LayerNorm}\left({V^{\prime }}_{R}^{l}+\mathrm{FFN}\left({V^{\prime }}_{R}^{l}\right)\right),$$

(12) $$V_{G}^{l+1}=\mathrm{LayerNorm}\left({V^{\prime }}_{G}^{l}+\mathrm{FFN}\left({V^{\prime }}_{G}^{l}\right)\right).$$

By stacking *N* such blocks, the proposed GFIM progressively promotes deeper interaction and fusion between region and grid features. As a result, the final visual representations can simultaneously preserve the object-centric semantic abstraction provided by region features and the fine-grained contextual details captured by grid features, thereby yielding more comprehensive and discriminative visual representations.

### 3.3. Text-Guided Feature Calibration Module

With the proposed Global-Guided Feature Interaction Module, we exploit the complementary properties of region and grid features to learn more comprehensive visual representations. Nevertheless, feature redundancy remains a non-negligible issue. Since both feature types are derived from the same image, they inevitably share overlapping information, while also containing background noise irrelevant to the query text, such as grass, sky, and other contextual content. To alleviate this problem, we further introduce a Text-Guided Feature Calibration Module, which employs auxiliary descriptions generated by a large language model as semantic priors to refine the fused visual features. By suppressing redundant and text-irrelevant information, the proposed module helps narrow the semantic gap between visual and textual modalities.

Specifically, given an image, we use LLaVA [44] as the image caption generator. LLaVA is a large-scale vision-language model that integrates visual perception with the language generation ability of large language models, enabling it to produce detailed and semantically rich descriptions for input images. To generate auxiliary textual descriptions, we employ the following prompt: “Describe this image in great detail. Focus on the main subjects, their actions, clothing, and spatial relationships. Also describe the background environment, lighting, and the overall atmosphere of the scene.”

Formally, the image caption generation process is defined as

(13) $$T_{caption}=\mathrm{LLaVA}\left(I,p\right),$$

where $t_{ic}$ denotes the generated image caption. We then feed $t_{ic}$ into the CLIP [45] text encoder, followed by a fully connected layer, to project it into a high-dimensional semantic space.

(14) $$t_{ic}=\mathrm{FC}\left({\mathrm{CLIP}}_{\mathrm{text}}\left(T_{caption}\right)\right).$$

After obtaining the image caption representation $t_{ic}$, we introduce a Text-Guided Feature Calibration Module, where $t_{ic}$ is used as the query, and the fused region features $V_{R}^{N}$ or fused grid features $V_{G}^{N}$ are used as the key and value. In this way, the semantic information conveyed by the generated caption guides the model to attend selectively to visual contents that are more relevant to the textual description. As a result, redundant or noisy information in the fused visual features can be effectively suppressed, enabling the model to emphasize semantically salient objects and thus produce more discriminative visual representations.

Formally, the calibration process is defined as

(15) $$v_{f}^{R}=\mathrm{MCA}\left(t_{ic},V_{R}^{N},V_{R}^{N}\right),$$

(16) $$v_{f}^{G}=\mathrm{MCA}\left(t_{ic},V_{G}^{N},V_{G}^{N}\right),$$

where MCA(·) follows the same standard multi-head cross-attention formulation as that used in the GFIM module, except that the query is replaced by the caption representation $t_{ic}$.

Nevertheless, such a calibration process may also remove weak but potentially useful information. To mitigate this issue, we further introduce a residual shortcut connection. Specifically, we first apply average pooling over the fused region features (or grid features) to obtain a global visual embedding, which is subsequently combined with the calibrated features via element-wise addition:

(17) $$v_{res}^{R}=v_{f}^{R}+z_{r},\;z_{r}=\mathrm{AvgPool}\left(V_{R}^{N}\right),$$

(18) $$v_{res}^{G}=v_{f}^{G}+z_{g},\;z_{g}=\mathrm{AvgPool}\left(V_{G}^{N}\right),$$

where $v_{c}^{R}$ and $v_{c}^{G}$ denote the context-enriched region and grid representations, respectively.

### 3.4. Adaptive Gating Fusion Module

Although $v_{res}^{R}$ and $v_{res}^{G}$ provide complementary visual cues, directly summing them may be suboptimal, since it implicitly assumes that region and grid representations contribute equally across all visual instances. In practice, however, their relative importance often varies with image content. Region features are generally more effective at emphasizing object-centric semantics, whereas grid features are better at preserving holistic spatial context. Therefore, a fixed fusion strategy may be insufficient to fully exploit their complementary strengths.

To achieve more flexible and instance-adaptive fusion, we further introduce an adaptive gating fusion module. Specifically, we first concatenate $v_{c}^{R}$ and $v_{c}^{G}$, and then feed the concatenated representation into a fully connected layer followed by a sigmoid activation function to generate a gating coefficient:

(19) $$\alpha =\sigma \left(\mathrm{FC}\left([v_{c}^{R};v_{c}^{G}]\right)\right),$$

where [·;·] denotes vector concatenation, FC(·) denotes a fully connected layer, and σ(·) denotes the sigmoid function. The gating coefficient α∈[0,1] is then used to adaptively balance the contributions of the two context-enriched visual representations:

(20) $$v=\alpha v_{c}^{R}+\left(1-\alpha \right)v_{c}^{G}.$$

Through this adaptive gating mechanism, the model can dynamically emphasize the more informative feature source while suppressing the less useful one according to the content of each image. In this way, object-level semantic cues and global spatial context can be more effectively integrated, leading to a more robust and discriminative final visual representation.

### 3.5. Loss Function

In line with previous studies [5,29], we employ the bidirectional triplet ranking loss [3] to optimize the proposed model. The main objective is to ensure that the similarity of a matched Image–Text pair is higher than that of any mismatched pair by at least a predefined margin. Formally, the loss function is defined as

(21) $$L={\left[\lambda -S\left(v,t\right)+S\left(\hat{v},t\right)\right]}_{+}+{\left[\lambda -S\left(v,t\right)+S\left(v,\hat{t}\right)\right]}_{+},$$

where λ denotes the margin hyperparameter, which specifies the minimum required gap between positive and negative pairs in the embedding space. The operator ${\left[x\right]}_{+}\equiv \max\left(x,0\right)$ represents the hinge function. *S* denotes the similarity between an image and a text, which is implemented as cosine similarity in our experiments. In addition, $\hat{v}$ and $\hat{t}$ represent the hardest negative image and text samples within a mini-batch, respectively, and are defined as: ${\hat{v}}^{\prime }=\underset{x\neq v^{\prime }}{\arg\max}S\left(x,t^{\prime }\right)$ and ${\hat{t}}^{\prime }=\underset{y\neq t^{\prime }}{\arg\max}S\left(v^{\prime },y\right)$.

## 4. Experiments

### 4.1. Datasets and Evaluation Metrics

#### 4.1.1. Datasets

Following previous works [5,10,11], we evaluate our approach on the standard Flickr30K [46] and MS-COCO [47] benchmarks. Each image in both datasets is annotated with five captions, which provide diverse textual descriptions for Image–Text matching. For Flickr30K, we use the standard split of 29,000 images for training, 1000 for validation, and 1000 for testing. For MS-COCO, we follow the commonly adopted split containing 113,287 training images, 5000 validation images, and 5000 test images. As in prior work, results on MS-COCO are reported under two settings: (1) MS-COCO 1K, where the 5000 test images are divided into five folds of 1,000 images and the average result over the five folds is reported; and (2) MS-COCO 5K, where evaluation is performed on the entire 5000-image test set.

#### 4.1.2. Evaluation Metrics

For fair comparison with existing methods, we adopt two widely used evaluation metrics in cross-modal Image–Text retrieval, namely Recall@K (R@K) and Recall Sum (rSum), to assess model performance. Specifically, Recall@K measures the percentage of queries for which at least one correct match is retrieved within the top-*K* ranked results, where a higher value indicates better retrieval performance. Following previous works [5,48], we report R@1, R@5, and R@10. To measure the overall retrieval performance, we further report rSum. Since Image–Text retrieval is a bidirectional task, rSum is computed as the sum of the six recall scores from both image-to-text and text-to-image retrieval:

(22) $$\mathrm{rSum}=\underset{Image\text{-}to\text{-}Text}{\underset{︸}{\left(R@1+R@5+R@10\right)}}+\underset{Text\text{-}to\text{-}Image}{\underset{︸}{\left(R@1+R@5+R@10\right)}},$$

where the first three terms correspond to the recall scores for image-to-text retrieval, and the last three terms correspond to those for text-to-image retrieval. A higher rSum indicates better overall cross-modal retrieval performance.

### 4.2. Implementation Details

For the visual branch, we use the BUTD [8] model pretrained on the VG [49] dataset to extract region features, and employ ViT [15] or Swin Transformer [16] to obtain grid features. For the textual branch, we use a pretrained BERT [43] model to extract word features. For grid feature extraction, the input image resolution is set to 224 × 224 or 384 × 384, yielding 14 × 14 and 24 × 24 grids for ViT, and 7 × 7 and 12 × 12 grids for Swin Transformer, respectively. To generate detailed image descriptions, we employ LLaVA [44], followed by the CLIP [45] text encoder to extract global caption features. During training, the BUTD model, LLaVA, and the CLIP text encoder are kept frozen. The model is trained for 25 epochs using the AdamW optimizer with a weight decay of 0.0001. The initial learning rate is 0.0005 and is decayed by a factor of 10 at the 9th, 15th, 20th, and 25th epochs. The batch size is set to 128, the shared embedding dimension *d* is 1024, and the triplet loss margin λ is 0.2. All experiments are conducted on two NVIDIA 5090 GPUs.

### 4.3. Comparisons with State-of-the-Art Methods

We compare the proposed method with a broad range of representative cross-modal Image–Text retrieval approaches. According to the adopted visual representations, these methods can be roughly divided into three categories: (1) grid-feature-based methods, including VSE++ [3], SCO [50], GXN [51], LAPS [10], PICO [14], CDDS [12], AVSE [11], and MG-Net [13]; (2) region-feature-based methods, including SCAN [5], VSRN [4], CAAN [52], GSMN [53], NAAF [29], CHAN [28], IMEB [2], TVRN [1], RVSE++ [22], Hire [31], and SSCT [27]; and (3) methods that jointly exploit grid and region features, such as DSRAN [17], TWO [18], and MVFFN [19].

Table 1 reports the quantitative comparison between the proposed model and the aforementioned cross-modal Image–Text retrieval methods on two public benchmarks, Flickr30K and MS-COCO. As can be seen, our method achieves highly competitive performance on almost all evaluation metrics and obtains the best overall results in terms of rSum under the reported settings. These results demonstrate the effectiveness of the proposed method for cross-modal Image–Text retrieval. Specifically, on the Flickr30K dataset, when adopting ViT-Base-224 as the backbone for grid feature extraction, our method achieves R@1 scores of 84.3% and 69.1% for image-to-text and text-to-image retrieval, respectively. When a stronger ViT-Base-384 backbone is used, the corresponding R@1 scores are further improved to 84.7% and 71.5%, respectively. Compared with the best competing method that combines grid and region features, namely MVFFN [19], our method improves the R@1 accuracy from 84.0% to 84.7% for image-to-text retrieval, and from 65.5% to 71.5% for text-to-image retrieval, respectively. Compared with the best grid-feature-based method, namely MG-Net [13], our method also shows clear superiority, achieving gains of 5.4% and 7.7% in R@1 for image-to-text and text-to-image retrieval, respectively. In terms of the overall recall metric rSum, our method achieves 536.6 and 541.0, which surpass the previous best method MVFFN (527.4) by a clear margin. These results further verify the advantage of the proposed method in learning more effective bidirectional cross-modal representations.

Similarly, strong retrieval performance is consistently observed on both the MS-COCO 1K and MS-COCO 5K test sets. In particular, with ViT-Base-384 as the visual backbone, our method achieves R@1 scores of 82.3% and 69.7% for image-to-text and text-to-image retrieval, respectively, on the MS-COCO 1K test set. Although its image-to-text R@1 is slightly lower than that of MVFFN (82.7%), it attains a higher text-to-image R@1 and the best overall rSum of 538.6, surpassing MVFFN (537.5). On the MS-COCO 5K test set, our method likewise improves the overall retrieval performance, exceeding MVFFN by 3.6% in terms of rSum. These results further verify the effectiveness and robustness of the proposed method across different datasets and evaluation settings.

Additionally, we conduct experiments with other commonly used visual backbones, as shown in Table 2. Specifically, we use Swin-Base as the visual backbone to extract grid features with input resolutions of 224 and 384. Under the same input resolution, Swin-Base consistently achieves better performance than ViT in our experiments. We believe this is because Swin adopts a hierarchical architecture with shifted-window attention, which is more effective at preserving local structures and multi-scale information, thereby providing more suitable visual representations for our grid-region feature fusion framework. The results show that our method maintains competitive performance across different backbone architectures, demonstrating its adaptability to diverse visual encoders. Although the rSum under the Swin-Base-384 setting is slightly lower than that of CDDS [12], the overall results remain encouraging.

Overall, the above results can be understood from the complementary roles of region-level and grid-level visual representations. Region features are more naturally aligned with object-centric textual semantics, while grid features preserve richer contextual and spatial information that is important for capturing small objects, background cues, and global scene structure. By jointly exploiting these two types of visual information, the proposed method can achieve more comprehensive cross-modal alignment and thus improve bidirectional Image–Text retrieval. The consistent gains across different benchmarks and evaluation settings further indicate that the effectiveness of the proposed method is not due to a specific dataset or metric, but comes from a more principled integration of fine-grained semantic cues and holistic contextual representations.

### 4.4. Ablation Studies

To further validate the effectiveness of the proposed model, we conduct comprehensive ablation experiments. Unless otherwise specified, all experiments are performed on the Flickr30K test set with ViT-Base-224 as the visual backbone.

#### 4.4.1. Component Analysis

Table 3 presents the ablation results of different key components in the proposed model. It can be observed that using grid features alone achieves better performance than using region features alone, while their combination leads to better performance than using either of them alone. Specifically, the Region+Grid baseline achieves an rSum of 516.3, which is higher than that of using only region features (501.1) or only grid features (506.5). This demonstrates that the two types of visual features are complementary. On the basis of the Region+Grid baseline, we further evaluate the contributions of GFIM, TFCM, and AGFM. When GFIM is added, the rSum increases from 516.3 to 519.5, showing that modeling interactions between different visual features is beneficial. When only TFCM is introduced, the rSum reaches 533.1, indicating that the semantic priors provided by auxiliary image descriptions can effectively filter out redundant and text-irrelevant visual information. Similarly, AGFM also brings performance gains, improving the rSum to 527.9. Moreover, combining multiple components leads to further improvements. For example, jointly using GFIM and TFCM increases the rSum to 535.6, which is better than using either module alone. Finally, when all three modules are integrated, the full model achieves the best overall performance with an rSum of 536.6. These results verify that cross-feature interaction, semantic calibration, and adaptive fusion are all important for learning more comprehensive and discriminative visual representations.

#### 4.4.2. Analysis of the GFIM

In this subsection, we compare two commonly used visual feature fusion architectures, with the results reported in Table 4. Structure 1 encodes region features and grid features using two independent branches and directly fuses their outputs by element-wise addition. Structure 2 further introduces self-attention to model intra-feature interactions and cross-attention to capture inter-feature interactions. As shown in Table 4, both structures perform worse than the proposed method, with rSum drops of 5.7% and 9.3%, respectively. The inferior performance of Structure 1 is likely due to the insufficient interaction between region and grid features. Although Structure 2 employs more complex attention-based interactions, its performance degrades further, suggesting that simply increasing interaction complexity cannot guarantee effective fusion and may even introduce semantic noise without proper guidance. In contrast, GFIM performs bidirectional interaction between region and grid features under global semantic guidance. This design enhances cross-feature dependency modeling and improves semantic consistency during the fusion process. Consequently, GFIM learns more complementary visual representations and achieves superior retrieval performance.

Moreover, we further investigate the effect of the number of stacked GFIM blocks *N*. As reported in Table 5, N=1 achieves the best result, with an rSum of 536.6, while increasing *N* to 2 and 3 decreases the rSum to 531.0 and 521.8, respectively. This suggests that a shallow interaction design is sufficient for modeling the complementarity between region and grid features. In contrast, stacking more GFIM blocks may cause over-interaction between the two feature streams, resulting in redundant information propagation and reduced feature diversity. Consequently, the model becomes less effective in preserving the distinct advantages of object-centric semantics and contextual spatial cues, which leads to degraded retrieval performance.

#### 4.4.3. Analysis of the AGFM

To further evaluate the effectiveness of AGFM, we compare it with two alternative gating strategies in Table 6, including a concatenation-MLP variant and a sample-wise softmax variant. Both alternatives perform worse than AGFM, with rSum drops of 20.1% and 3.6%, respectively. The substantially inferior performance of the concatenation-MLP variant may stem from its implicit and coarse fusion manner. By directly concatenating region and grid features and feeding them into an MLP, the model lacks explicit branch-wise weighting and has difficulty accurately modeling the relative importance of the two heterogeneous feature types. This may also weaken their inherent structural and semantic complementarity, leading to redundant information and noisy feature interactions. In contrast, the softmax-based variant explicitly models branch-wise importance, but its competitive normalization may overly suppress one branch and thus limit feature complementarity. By comparison, AGFM adopts a sigmoid-based gating mechanism for soft and adaptive fusion, enabling a more balanced integration of object-level semantics and global spatial context, thereby achieving better retrieval performance.

#### 4.4.4. Effect of Auxiliary Text Quality

To investigate the influence of auxiliary text quality, we replace LLaVA with BLIP2 as the auxiliary description generator. As shown in Table 7, using BLIP2-generated descriptions achieves R@1 scores of 82.5% and 67.7% for image-to-text and text-to-image retrieval, respectively, with an rSum of 530.2. Compared with LLaVA, the performance decreases to some extent, indicating that higher-quality auxiliary descriptions can provide more effective semantic guidance for TFCM. Nevertheless, the model still maintains competitive performance with BLIP2, suggesting that the proposed framework is not overly dependent on a specific generator. Since auxiliary descriptions are used as semantic guidance rather than strict supervision, TFCM shows a certain degree of robustness to noisy or incomplete descriptions. However, substantial semantic errors may weaken the guidance and lead to performance degradation.

### 4.5. Qualitative Results

**Figure 4:**
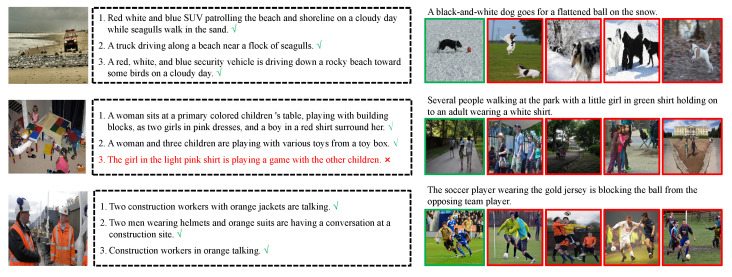
Qualitative results on the Flickr30K dataset. For each image query, we visualize the top three retrieved sentences, where correct and incorrect results are marked with green checkmarks and red crosses, respectively. For each text query, we show the top five retrieved images, with correct and incorrect results highlighted by green and red boxes, respectively.

To further assess the proposed method, Figure 4 provides qualitative examples of bidirectional retrieval. For each image query, the top three retrieved sentences are presented, where correct and incorrect matches are marked with green checkmarks and red crosses, respectively. For each text query, the top five retrieved images are shown, with correct and incorrect matches indicated by green and red boxes, respectively.

The results suggest that combining region and grid features helps the model capture visual content more completely, which in turn leads to better Image–Text alignment. For example, in the first image query, the model not only identifies the main object, i.e., the vehicle, but also captures informative details such as the seagulls as well as the surrounding beach scene. This allows it to retrieve sentences that are more consistent with the image semantics. In addition, with the aid of Text-Guided semantic calibration, the model can rank the correct image at the top even when many candidates share similar visual patterns, as shown in the third text query. Overall, these qualitative results provide further evidence that the proposed method is effective in modeling complementary visual cues and improving cross-modal retrieval.

### 4.6. Feature Visualization Analysis

**Figure 5:**
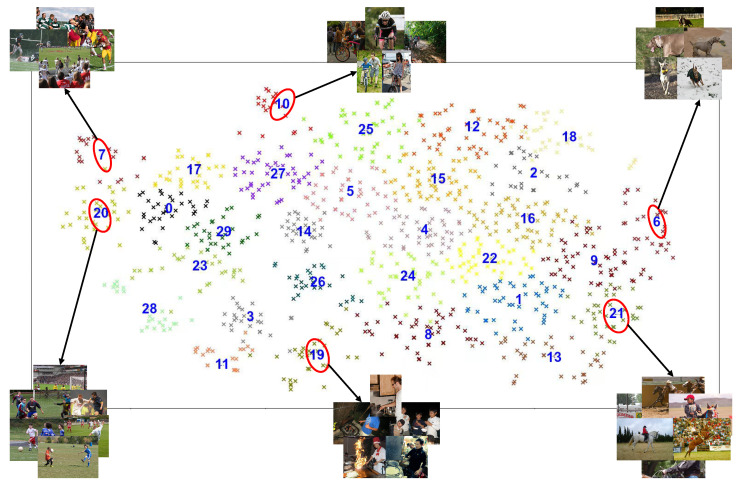
T-SNE visualization of the learned visual features. The features are partitioned into 30 clusters by K-Means, with colors distinguishing different clusters and numbers denoting the cluster labels at their corresponding centroids.

To intuitively analyze the learned representation space, we visualize the final visual features using T-SNE. The high-dimensional visual embeddings are first projected into a two-dimensional space and then grouped into 30 clusters using K-Means. Different colors denote different clusters, and each cluster centroid is annotated with its corresponding index. Figure 5 shows the visualization results and representative samples from selected clusters.

It can be observed that samples within the same cluster generally exhibit similar semantic content, whereas samples from different clusters are well separated in the feature space. For instance, cluster 10 mainly consists of cycling-related images, suggesting that the learned representations can effectively aggregate samples with similar visual and semantic characteristics. By contrast, cluster 19, which mainly corresponds to cooking scenes, is clearly separated from cluster 6, which contains dog-related images, indicating that the model can distinguish categories with substantial semantic differences. Moreover, although clusters 7 and 20 both contain ball-sport-related images, such as rugby and football, they still form distinguishable groups in the feature space. This observation shows that the model can capture not only high-level semantic similarities but also fine-grained variations among semantically related categories. Overall, these visualization results indicate that the learned visual features are both semantically coherent and discriminative, further confirming the effectiveness of our model.

## 5. Conclusions

This paper presents a feature fusion network for Image–Text retrieval, aiming to better exploit the complementary advantages of region features and grid features. To this end, we propose a Global-Guided Feature Interaction Module to enhance information exchange between the two types of visual features, a Text-Guided Feature Calibration Module to reduce redundant and text-irrelevant visual content with the help of auxiliary image descriptions, and an Adaptive Gating Fusion Module to dynamically combine multiple visual representations. By integrating visual interaction, semantic calibration, and adaptive fusion into a unified framework, the proposed method learns more informative and discriminative visual features for cross-modal matching. Extensive experiments on MS-COCO and Flickr30K demonstrate the effectiveness of our method and its competitive performance compared with recent state-of-the-art approaches. In future work, we will investigate more efficient feature interaction strategies and stronger multimodal representation learning frameworks to further improve retrieval performance.

## Figures and Tables

**Table 1:** Comparison of experimental results on the MS-COCO and Flickr30K datasets using ViT-Base [15] and Swin Transformer [16] as backbones. The best results are highlighted in bold.

Methods	Feature Type	Flickr30K 1K Test							MS-COCO 1K Test							MS-COCO 5K Test						
		Image-to-Text			Text-to-Image			rSum	Image-to-Text			Text-to-Image			rSum	Image-to-Text			Text-to-Image			rSum
		R@1	R@5	R@10	R@1	R@5	R@10		R@1	R@5	R@10	R@1	R@5	R@10		R@1	R@5	R@10	R@1	R@5	R@10	
VSE++ [3]	Grid	52.9	80.5	87.2	39.6	70.1	79.5	409.8	64.6	90.0	95.7	52.0	84.3	92.0	478.6	41.3	71.1	81.2	30.3	59.4	72.4	355.7
SCO [50]	Grid	55.5	82.0	89.3	41.1	70.5	80.1	418.5	69.9	92.9	97.5	56.7	87.5	94.8	499.3	42.8	72.3	83.0	33.1	62.9	75.5	369.6
GXN [51]	Grid	56.8	–	89.6	41.5	–	80.1	–	68.5	–	97.9	56.6	–	94.5	–	42.0	–	84.7	31.7	–	74.6	–
LAPS [10]	Grid	74.0	93.4	97.4	62.5	87.3	92.7	507.3	78.7	95.5	98.3	66.2	91.3	96.2	526.2	57.5	84.0	90.8	44.5	74.0	83.6	434.4
PICO [14]	Grid	74.5	94.0	98.2	63.0	88.5	93.1	511.3	78.8	95.9	98.8	66.3	91.6	96.5	527.9	57.5	84.1	91.2	44.9	74.3	83.8	435.8
CDDS [12]	Grid	74.8	93.6	97.8	63.1	88.2	93.1	510.6	79.0	95.9	98.7	66.5	91.8	96.8	528.7	57.9	84.6	91.4	45.0	74.8	84.1	437.8
AVSE [11]	Grid	76.0	94.6	97.5	62.7	88.4	93.1	512.3	79.8	95.6	98.3	67.0	91.5	96.3	528.5	58.8	84.3	91.0	45.1	74.3	83.9	437.4
MG-Net [13]	Grid	78.9	94.8	97.7	63.4	87.5	92.7	515.0	80.5	96.1	98.4	66.7	90.8	95.6	528.1	60.1	85.5	91.5	45.5	73.9	83.1	439.7
SCAN* [5]	Region	67.4	90.3	95.8	48.6	77.7	85.2	465.0	72.7	94.8	98.4	58.8	88.4	94.8	507.9	50.4	82.2	90.0	38.6	69.3	80.4	410.9
VSRN* [4]	Region	71.3	90.6	96.0	54.7	81.8	88.2	482.6	76.2	94.8	98.2	62.8	89.7	95.1	516.8	53.0	81.1	89.4	40.5	70.6	81.1	415.7
CAAN [52]	Region	70.1	91.6	97.2	52.8	79.0	87.9	478.6	75.5	95.4	98.5	61.3	89.7	95.2	515.6	52.5	83.3	90.9	41.2	70.3	82.9	421.1
GSMN* [53]	Region	76.4	94.3	97.3	57.4	82.3	89.0	496.7	78.4	96.4	98.6	63.3	90.1	95.7	522.5	–	–	–	–	–	–	–
NAAF [29]	Region	81.9	96.1	98.3	61.0	85.3	90.6	513.2	80.5	96.5	98.8	64.1	90.7	96.5	527.2	58.9	85.2	92.0	42.5	70.9	81.4	430.9
CHAN [28]	Region	80.6	96.1	97.8	63.9	87.5	92.6	518.5	81.4	96.9	98.9	66.5	92.1	96.7	532.5	59.8	87.2	93.3	44.9	74.5	84.2	443.9
IMEB [2]	Region	84.2	96.7	98.4	64.0	88.0	92.8	524.1	82.4	96.9	99.0	66.7	91.9	96.6	533.5	62.8	87.8	93.5	44.9	74.6	84.0	447.6
TVRN [1]	Region	82.1	95.6	98.3	63.9	87.6	92.6	520.1	81.1	96.4	98.8	67.7	92.3	97.1	533.4	61.1	86.3	92.5	45.5	75.0	84.8	445.2
RVSE++ [22]	Region	83.2	96.0	98.2	63.1	86.9	92.1	519.5	78.1	96.3	98.8	62.1	90.3	95.8	521.4	59.8	85.8	92.3	43.6	73.4	83.6	438.5
Hire [31]	Region	83.0	97.0	98.8	65.9	89.1	93.4	527.1	81.6	96.6	99.0	66.4	92.3	96.8	532.6	61.7	86.7	92.8	45.2	74.5	84.2	445.0
SSCT [27]	Region	82.3	96.0	98.6	62.5	86.5	91.5	517.5	81.9	97.1	98.8	66.4	91.5	96.5	532.1	62.2	87.2	93.3	45.1	74.0	83.6	445.4
DSRAN [17]	Grid+Region	80.5	95.5	97.9	59.2	86.0	91.9	511.0	80.6	96.7	98.7	64.5	90.8	95.8	527.1	57.9	85.3	92.0	41.7	72.7	82.8	432.4
TWO [18]	Grid+Region	82.1	96.2	98.5	64.7	88.4	93.1	523.0	81.5	96.8	99.0	66.6	92.2	96.8	532.9	61.1	86.6	92.8	44.9	74.2	83.7	443.3
MVFFN [19]	Grid+Region	84.0	96.9	98.8	65.5	88.6	93.6	527.4	82.7	97.0	99.0	68.5	93.2	97.1	537.5	62.4	87.5	94.0	47.3	75.0	85.6	451.8
**Ours(Vit-Base-224)**	Grid+Region	**84.3**	**97.9**	**99.1**	**69.1**	**91.2**	**95.0**	**536.6**	**80.9**	**97.4**	**99.2**	**69.1**	**92.7**	**96.5**	**535.8**	**60.2**	**87.4**	**93.7**	**46.2**	**76.0**	**85.3**	**448.8**
**Ours(Vit-Base-384)**	Grid+Region	**84.7**	**98.1**	**99.1**	**71.5**	**92.1**	**95.6**	**541.0**	**82.3**	**97.4**	**99.3**	**69.7**	**93.2**	**96.7**	**538.6**	**63.0**	**88.0**	**93.7**	**47.9**	**76.8**	**85.8**	**455.4**

Note: Results marked with an asterisk (*) were obtained using an ensemble of two models. Rows with a light-blue background indicate the results of the proposed method.

**Table 2:** Comparison of experimental results on Flickr30K dataset using Swin Transformer [16] as visual backbones. The best results are highlighted in bold.

Methods	Flickr30K 1K Test						
	Image-to-Text			Text-to-Image			rSum
	R@1	R@5	R@10	R@1	R@5	R@10	
***Swin-Base-224 + BERT-base, 7×7 patches***							
VSE++ [3]	82.5	96.5	98.9	70.0	91.4	95.1	534.4
SCAN [5]	79.0	95.9	98.2	67.7	90.6	94.9	526.3
LAPS [10]	82.4	97.4	99.5	70.0	91.7	95.4	536.4
PICO [14]	82.9	97.9	99.6	70.3	92.2	95.6	538.5
AVSE [11]	83.9	97.4	99.4	70.0	92.4	95.6	538.7
CDDS [12]	83.0	98.1	99.7	70.3	92.1	95.8	539.0
**Ours RGICN**	**86.2**	**97.3**	**99.5**	**72.4**	**93.0**	**96.3**	**544.7**
***Swin-Base-384 + BERT-base, 12×12 patches***							
VSE++ [3]	83.3	97.5	99.2	71.1	93.2	96.2	540.5
SCAN [5]	81.9	96.9	98.9	70.0	92.7	95.8	536.2
LAPS [10]	85.1	97.7	99.2	74.0	93.0	96.3	545.3
PICO [14]	85.8	98.1	99.4	74.5	93.5	96.9	548.2
AVSE [11]	87.1	98.3	99.2	73.6	93.5	96.5	548.2
CDDS [12]	86.8	98.3	99.6	76.3	94.3	97.2	552.5
**Ours RGICN**	**87.1**	**98.3**	**99.7**	**74.3**	**93.9**	**96.7**	**550.1**

**Table 3:** Ablation experiments for each key component. The best results are highlighted in bold.

Methods	Model			Image-to-Text			Text-to-Image			rSum
	GFIM	TFCM	AGFM	R@1	R@5	R@10	R@1	R@5	R@10	
Region	✗	✗	✗	76.5	93.6	97.5	58.3	84.5	90.7	501.1
Grid	✗	✗	✗	75.5	93.6	97.6	61.1	86.4	92.4	506.5
	✗	✗	✗	78.0	96.0	98.4	63.2	87.7	93.0	516.3
	✓	✗	✗	80.2	95.6	98.1	64.2	98.2	93.3	519.5
Region+Grid	✗	✓	✗	82.5	96.7	98.9	68.3	91.4	95.3	533.1
	✗	✗	✓	82.6	96.7	99.2	65.9	89.4	94.2	527.9
	✓	✓	✗	83.7	97.4	99.3	68.8	91.3	95.2	535.6
**RGICN**	✓	✓	✓	**84.3**	**97.9**	**99.1**	**69.1**	91.2	95.0	**536.6**

Note: A checkmark (✓) indicates that the corresponding component is used; a cross (✗) indicates that it is not used. Rows with a light-blue background indicate the results of the proposed method.

**Table 4:** Ablation study on different visual feature fusion architectures. The best results are highlighted in bold.

Methods	Image-to-Text			Text-to-Image			rSum
	R@1	R@5	R@10	R@1	R@5	R@10	
Structure1	82.1	96.5	98.5	67.7	91.1	95.0	530.9
Structure2	81.8	96.3	98.6	66.8	89.7	94.1	527.3
**Ours GFIM**	**84.3**	**97.9**	**99.1**	**69.1**	**91.2**	**95.0**	**536.6**

Note: Rows with a light-blue background indicate the results of the proposed method.

**Table 5:** Ablation study on the number of stacked GFIM blocks. The best results are highlighted in bold.

Methods	Image-to-Text			Text-to-Image			rSum
	R@1	R@5	R@10	R@1	R@5	R@10	
N = 3	80.1	94.7	98.0	65.7	89.4	93.9	521.8
N = 2	81.8	97.1	99.5	67.2	90.5	94.8	531.0
**N = 1**	**84.3**	**97.9**	**99.1**	**69.1**	**91.2**	**95.0**	**536.6**

Note: Rows with a light-blue background indicate the results of the proposed method.

**Table 6:** Ablation study on different fusion strategies. The best results are highlighted in bold.

Methods	Image-to-Text			Text-to-Image			rSum
	R@1	R@5	R@10	R@1	R@5	R@10	
Concat-MLP	77.4	94.6	97.8	63.5	89.3	93.9	516.5
Sample-wise Softmax	82.6	97.7	99.0	68.1	90.9	94.7	533.0
**Ours AGFM**	**84.3**	**97.9**	**99.1**	**69.1**	**91.2**	**95.0**	**536.6**

Note: Rows with a light-blue background indicate the results of the proposed method.

**Table 7:** Ablation study on different auxiliary description generators. The best results are highlighted in bold.

Methods	Image-to-Text			Text-to-Image			rSum
	R@1	R@5	R@10	R@1	R@5	R@10	
BLIP2	82.5	97.6	99.0	67.7	89.4	94.0	530.2
**Ours LLaVA**	**84.3**	**97.9**	**99.1**	**69.1**	**91.2**	**95.0**	**536.6**

Note: Rows with a light-blue background indicate the results of the proposed method.

## Data Availability

The datasets used in this study are publicly available benchmark datasets, including Flickr30K and MS-COCO.
